# The bacterial communities of Alaskan mosses and their contributions to N_2_-fixation

**DOI:** 10.1186/s40168-021-01001-4

**Published:** 2021-02-23

**Authors:** Hannah Holland-Moritz, Julia E. M. Stuart, Lily R. Lewis, Samantha N. Miller, Michelle C. Mack, Jose Miguel Ponciano, Stuart F. McDaniel, Noah Fierer

**Affiliations:** 1 Department of Ecology and Evolutionary Biology, University of Colorado, Boulder, CO USA; 2 Center for Ecosystem Science and Society and the Department of Biological Sciences, Northern Arizona University, Flagstaff, AZ USA; 3 Provost’s Office, University of Florida, Gainesville, FL USA; 4 Department of Biology, University of Florida, Gainesville, FL USA; 5 Cooperative Institute for Research in Environmental Sciences, University of Colorado at Boulder, Boulder, CO USA

**Keywords:** Bryophytes, Plant microbiome, Phyllosphere, Nitrogen fixation

## Abstract

**Background:**

Mosses in high-latitude ecosystems harbor diverse bacterial taxa, including N_2_-fixers which are key contributors to nitrogen dynamics in these systems. Yet the relative importance of moss host species, and environmental factors, in structuring these microbial communities and their N_2_-fixing potential remains unclear. We studied 26 boreal and tundra moss species across 24 sites in Alaska, USA, from 61 to 69° N. We used cultivation-independent approaches to characterize the variation in moss-associated bacterial communities as a function of host species identity and site characteristics. We also measured N_2_-fixation rates via ^15^N_2_ isotopic enrichment and identified potential N_2_-fixing bacteria using available literature and genomic information.

**Results:**

Host species identity and host evolutionary history were both highly predictive of moss microbiome composition, highlighting strong phylogenetic coherence in these microbial communities. Although less important, light availability and temperature also influenced composition of the moss microbiome. Finally, we identified putative N_2_-fixing bacteria specific to some moss hosts, including potential N_2_-fixing bacteria outside well-studied cyanobacterial clades.

**Conclusions:**

The strong effect of host identity on moss-associated bacterial communities demonstrates mosses’ utility for understanding plant-microbe interactions in non-leguminous systems. Our work also highlights the likely importance of novel bacterial taxa to N_2_-fixation in high-latitude ecosystems.

Video Abstract

**Supplementary Information:**

The online version contains supplementary material available at 10.1186/s40168-021-01001-4.

## Introduction

Mosses are ubiquitous in northern ecosystems, often forming the dominant ground cover in boreal forests, peatlands, and Arctic tundra [1]. Mosses play critical ecological roles in these high-latitude environments as they insulate soils, maintain high soil moisture, and mediate carbon (C) and nitrogen (N) cycles [2]. Moss growth typically represents 20–50% of net primary productivity [2], and mosses are an important source of biologically fixed N in high-latitude ecosystems [3, 4]. Mosses also typically have high N-use efficiencies and low decomposition rates that promote C and N accumulation in many high-latitude ecosystems [2, 5, 6].

Like all plants, mosses associate with microbes and these microbial communities have key roles in multiple ecosystem-level processes. Moss-associated microbes fix N_2_ [3, 4, 7], oxidize methane [8, 9], and contribute to the decomposition of organic matter in moss-dominated tundra and peatlands [10]. For these reasons, there has long been an interest in understanding what microbes associate with mosses, their contributions to ecosystem processes, and the factors that structure moss-associated microbial communities (e.g., [11–14]).

Moss microbiomes are characterized by a core community of bacteria that are typically abundant in many common boreal and tundra mosses [13, 15]. The moss microbiome is often highly host specific, with different moss species harboring distinct bacterial communities [15, 16]. However, host identity is not the sole factor determining moss microbiome composition as there is clearly some degree of variation in the moss microbiome across a given species, variation that is presumed to be related to local environmental conditions [17, 18].

More generally, the relative importance of environment versus host species in mediating moss microbiome composition remains unresolved. With our previous work [15], we found environment to have a negligible effect on microbial community structure compared to moss species identity; however, the moss samples included in that study were collected from a single 4 km^2^ area. It is likely that over a larger geographic area, spanning broader environmental gradients (i.e., latitudinal climate shifts and forest versus tundra ecosystem types), site conditions could play a more prominent role in structuring microbial communities. Likewise, although moss microbial communities appear to be strongly influenced by host identity, it is unclear whether the differences in microbial community composition can be predicted from host phylogenetic relationships, the role of host species effects relative to geographic effects, and importantly, whether the composition of the microbial communities found in a given host species is stable across broad environmental gradients.

These questions are not unique to the study of moss microbiomes, but the important role of bacterial N_2_-fixation makes the moss microbiome a useful model system for simultaneously studying plant-microbe associations and how these associations influence N cycling in high-latitude systems. The importance of moss-associated N_2_-fixing bacteria has been recognized for years [3, 19, 20] and numerous studies have focused specifically on the role of cyanobacteria, particularly *Nostoc*, in N_2_-fixation [4, 21–25]. However, several studies suggest that cyanobacteria may not be the only N_2_-fixing microbes associated with mosses [26, 27], but the relative importance of these non-cyanobacterial N_2_-fixers and their occurrence across different moss species has not been well-documented. Information on the distributions of those bacteria capable of N_2_-fixation in mosses and their relative contributions to N_2_-fixation rates can be used to better predict how the capacity for N_2_-fixation varies across hosts and environmental gradients.

The environmental factors structuring the microbial communities of common high-latitude mosses remain largely undetermined. However, warming experiments in *Sphagnum* bogs suggest that temperature regime likely plays a role in structuring the moss microbiome [14]. Light availability and moisture have also been shown to be important in the activity of moss-associated cyanobacteria [4, 28, 29], and these factors may also have direct effects on other microbial taxa. Alternatively, light and moisture may indirectly affect abundances of other microbial taxa as a result of higher N availability provided by the light and moisture sensitive N_2_-fixing cyanobacteria. Finally, soil pH may be yet another environmental factor structuring microbial communities in these host-associated systems [30].

With this study we sought to identify the factors structuring moss-associated bacterial communities and their potential contributions to N_2_-fixation. We asked three questions: (1) Are environmental factors or host phylogeny more important in structuring the microbiomes of Alaskan mosses? (2) What factors structure the composition of moss microbiomes across broad environmental gradients? And (3) which particular moss-associated microbes are most likely responsible for the observed high N_2_-fixation rates? To address these questions, we collected samples of 26 common boreal moss species from 24 sites spanning a latitudinal transect in Alaska, USA (from 61 to 69° N). We used 16S rRNA gene sequencing to compare microbial community composition across host species and compared the relative effects of host versus environmental factors in structuring microbial community composition. We also quantitatively measured N_2_-fixation rates via ^15^N_2_ isotopic enrichment in paired moss subsamples (work published in detail by Stuart et al. [31]). We then identified potential N_2_-fixing bacteria based on the associations between these taxa and measured N_2_-fixation rates, using publicly available genomic data to confirm their putative N_2_-fixation capabilities.

## Methods

### Site environmental data and sample collection

**Fig. 1:**
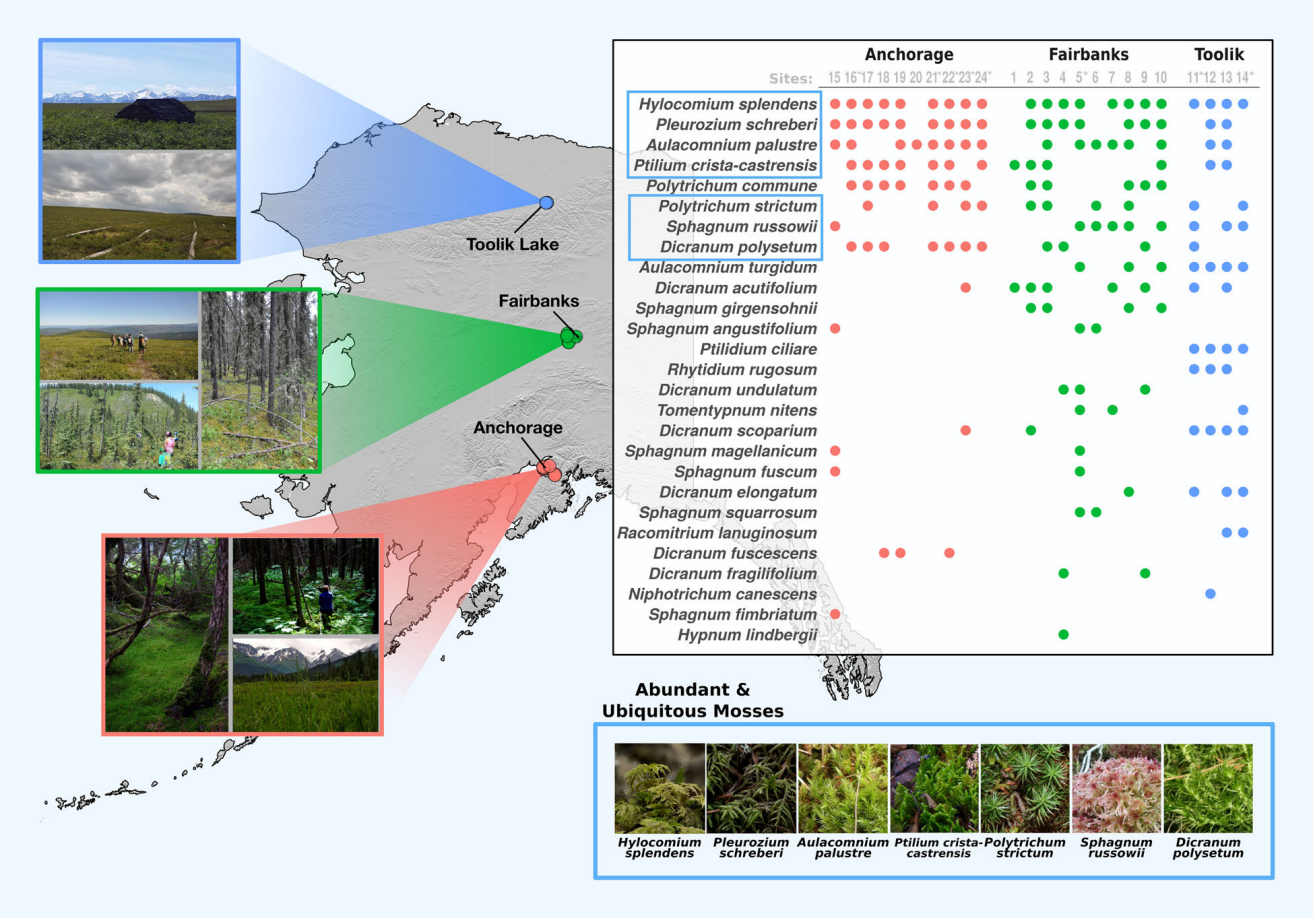
A map summarizing the species and sampling locations of the 461 samples in this study. Images (left) from each transect location show the environmental heterogeneity across the sites. The table (top right) displays the list of species sampled, ordered by the total number of samples collected for each species (see Supplementary Tables S1 and S2). Dots indicate the sites where at least one sample of a particular species was collected. The blue boxes indicate the seven moss species identified as “abundant and ubiquitous” in our data set (see the “Methods” section). Images of these seven species are displayed at the bottom right corner of the figure. The sites with the most moss species that were used in the site-specific analyses are marked with an asterisk

We collected microbial community marker gene and isotopic N_2_-fixation rate data from a total of 461 individual moss samples spanning 26 different moss species (see Table S1 for a full species list and Table S2 for details on what samples were collected from each site). These samples were collected from 24 sites across a latitudinal transect in Alaska, USA (from 61 to 69° N, Fig. 1). We selected sites that captured a breadth of common boreal and tundra habitat types in each of the three focal areas along the latitudinal transect (for more details about each site, see Table S3). We visited four sites near Toolik Lake, and 10 sites each near Fairbanks and Anchorage, AK, USA. We collected the samples over 2 years (2016 and 2017) with both collection periods occurring in late June. Site locations and the moss species collected at each site are summarized in Fig. 1.

At each site, we established a 30-m transect, with the latitude and longitude of the site (Table S3) corresponding to the mid-point of the transect. The transect was established to capture the breadth of environmental heterogeneity of each site. At each site, we conducted an informal field identification of the moss species present. Mosses were characterized as either locally common (appearing in 6 or more distinct patches at the site) or locally rare (appearing in 6 or fewer distinct patches). We collected at least 6 replicate samples of common mosses and as many replicates as were available for rare mosses from each site. Each sample consisted of 40 ramets taken from a colony (or patch) of clonal individuals and replicates were collected from separate patches. Due to the uncertain nature of field identification and the removal of some samples during sequencing quality control (see below), not every locally common species has exactly 6 replicates. Moss samples were collected randomly along the transect, with at least 5 m between replicates. For each sample, the nearest 5-m mark along the transect was noted and each sample was associated with the environmental data of the nearest measurement to its collection point.

Environmental data collected along the transect included soil pH, soil moisture, canopy cover, canopy composition, and canopy density. These environmental factors were chosen as they are typically used in the characterization of boreal and tundra vegetation communities or have been shown to be important in other studies of moss microbial communities [4, 28, 32]. Canopy cover was measured with a spherical densiometer, while canopy composition and density were determined by identifying the species of each tree within a 1 m radius of the 30-m transect and recording its basal area. This composition data was then converted into percent softwood and percent hardwood for each site. Soil pH was measured from a slurry created with a 2:1 ratio of water to soil. Soil moisture was measured by the change in wet to dried weight of biomass of a 5 × 5 × 5 cm plug of moss after 48 h in a drying oven at 60 °C (calculated as (wet weight-dry weight)/dry weight). We used the Worldclim database [33] to extract mean annual temperature and precipitation values corresponding to the latitude and longitude coordinates for each site (*n* = 24). As these values represent aggregated 30-year climate data at each site, they can be interpreted as the typical temperature and precipitation experienced at each site. Further details about site environmental characteristics are available in Stuart et al. [31].

For each sample, we collected moss from a mono-specific patch and stored samples in a cooler for less than 2 h until processed. After collecting each sample, we carefully cleaned off brown or decaying material with a gloved hand and sorted 40 ramets of approximately 5 cm in length into four equal subsamples (10 ramets each) for microbial sequencing, isotopic natural abundance, isotopic enrichment, and a museum voucher and genotyping specimen. When there were fewer than 40 ramets in a mono-specific patch, we placed five ramets each into the microbial and isotopic samples, leaving the remaining ramets for the voucher specimen. This strategy ensured that measurements of N_2_-fixation rate and microbial communities were as comparable as possible.

After sorting, we stored the moss samples for microbial analyses at − 20 °C prior to DNA extraction. Isotopic enrichment samples were stored in a cooler until measurements were started, usually within 2 h of sample sorting. Isotopic natural abundance samples and voucher specimens were dried at 60 °C for 48 h within 2 h of sorting.

### N_2_-fixation measurements

To measure the rates of N_2_-fixation for each of the 461 moss samples, we used an established isotopic enrichment protocol [31, 32]. Detailed results from the N_2_-fixation data associated with this study can be found in Stuart et al. [31]. Briefly, we lightly moistened each moss sample with one spray of ddH_2_O from a spray bottle and placed each moss sample into a 60-ml clear polycarbonate syringe that we filled with 10 ml of air and 10 ml of ^15^N_2_ (98% Atom enriched, Sigma-Aldrich Inc., USA) and sealed with a stopcock (for a total headspace of 20 ml). We incubated the sealed syringes at a natural outdoor location (a “common garden”) near the processing lab for 24 h to mimic the natural light and temperature conditions of the site from which the sample was collected. The common garden was centrally located for each sampling area (Anchorage, Fairbanks, and Toolik Lake) and similar to the collection sites in terms of light, temperature, and other environmental factors. Previous studies [34] have demonstrated the utility of this approach and have shown that ambient outdoor light changes are unlikely to have significant impact on the measured fixation rates in a common garden compared to the collection site [34, 35]. After 24 h, we removed the samples from the syringes and dried them in a 60 °C for 48 h. Natural abundance samples were immediately placed in a drying oven at 60 °C for 48 h and then shipped to Northern Arizona University for measurement of natural abundance of ^15^N and ^13^C.

N_2_-fixation rates were calculated by comparing the delta ^15^N values between the enriched and natural abundance samples. N concentrations from the enriched samples were then used to calculate the amount of N per dry moss weight. To measure these values, we ground each moss sample to a homogenous powder and analyzed it on a Costech ECS4010 coupled to a Thermo Scientific Delta V Advantage Isotope ratio mass spectrometer at Northern Arizona University. Calculations followed the method outlined in Jean et al. [32].

### Genotyping and moss phylogeny

For each moss species, we accessed previously published target capture DNA sequence data [36], using sequences from the liverwort *Ptilidium ciliare*, as an outgroup. Briefly, this method targets about 400 conserved plant exons for sequencing. We were unable to access data from the moss *Ptilium crista*-*castrensis*, so data from this species was not included in the phylogenetic analyses. We followed the bioinformatic pipeline outlined in Breinholt et al. [36] to generate a data matrix containing a single sequence for each locus for each species. We then concatenated all loci into a single supermatrix and used RAxML 8.2.10 with the GTR CAT model [37] to conduct a maximum likelihood search (100 nonparametric bootstrapping replicates) to generate a phylogeny. The resulting tree matched the phylogeny presented in Breinholt et al. [36], as well as phylogenies based on other data sources [38]. The samples used to construct the phylogenetic tree were archived at the University of Florida Herbarium (FLAS).

### Microbial 16S rRNA gene sequencing

To characterize the microbial communities found in each of the 461 moss samples, we used amplicon-based sequencing of a 253-bp region of the bacterial and archaeal 16S rRNA marker gene. First, we homogenized each sample (5 or 10 ramets per sample) with liquid N_2_ under aseptic conditions. We then extracted DNA from 0.25 g of homogenized tissue using the MoBio Power Soil DNA extraction kit (MoBio Laboratories, Carlsbad, CA). Following extraction, we used the 515f/806r primers to PCR amplify the V4-V5 region of the 16S rRNA gene [39]. Each sample was amplified using primer pairs that included a 12-bp barcode unique to each sample and Illumina sequencing adapters. Each sample was homogenized in a randomized order and was assigned a random location on 96-well plates during the DNA extraction and PCR amplification steps. Additionally, to control for any external contaminants, negative controls were included in both the extraction and PCR amplification steps. After triplicate PCR reactions, we normalized amplicon concentrations using ThermoFisher Scientific SequalPrep Normalization plates (Thermo Fisher Scientific Inc. USA) and pooled the amplicons together. Since samples were collected and processed over 2 years, they were sequenced in two batches. The first batch contained the 2016 samples, while the second contained all of the 2017 samples, samples that failed to sequence during the first run, and 50 randomly chosen duplicate samples from 2016 to quantify any potential run-to-run variation. We sequenced the pooled amplicons on the Illumina MiSeq platform with the 2 × 150 bp paired-end chemistry at the University of Colorado Next Generation Sequencing Facility. Raw sequence reads can be found at the GenBank SRA archive accession number PRJNA622832.

After sequencing, we demultiplexed samples using an in-house custom script [40] and used USEARCH v. 10 [41] to merge paired reads and filter low-quality sequences from the data (fastq_maxee_rate = 1.000). We used a single-nucleotide variant approach to create a de novo database of phylotypes in our samples. In this phylotype identification strategy, sequencing errors are first removed in a denoising step, and then phylotypes are assigned such that each phylotype is represented by a single unique sequence (i.e., all reads that are assigned to that phylotype are 100% identical). Our approach followed the UNOISE pipeline [42]. Briefly, we dereplicated the filtered and merged reads (“-fastx_uniques”) and then clustered sequences with the “-unoise3” command in USEARCH. We next filtered this database against the GreenGenes database (version August 2013 [43]) to remove phylotypes with a filter threshold of 75% similarity. We assumed sequences that did not meet this threshold were more likely to be of insufficient quality, chimeric, or a product of non-specific amplification.

Following the creation of the de novo database, we mapped the filtered and merged reads to the database to create a phylotype-by-sample table (USEARCH “otutab” command). We obtained between 3528 and 36,588 reads per sample, with an average sequencing depth of 16,495 reads per sample. We classified the reads against the GreenGenes database (version August 2013) using the RDP Naive Bayesian classifier with a minimum confidence threshold of 0.5 [44]. Chloroplasts and mitochondrial reads made up an average of 13% and 3.1%, respectively, of the total number of quality-filtered reads per sample and we removed these non-prokaryotic sequences from the table prior to downstream analyses. As in previous studies of moss microbial communities [13, 15], the majority of sequences were bacterial and no archaeal reads were detected in any of our samples.

After creating the phylotype abundance table, we performed several cleaning and quality control steps prior to downstream analyses. First, to control for differences in read depth across samples, we randomly selected 3000 reads per sample. This cut-off was chosen based on the read depth of the non-control sample (i.e., samples which were neither an extraction blank, nor a PCR no-template-control) with fewest reads after mitochondrial and chloroplast removal. Next, we compared our samples to blanks and filtered samples that were statistically indistinguishable from the blanks; samples that fell within a 97% ellipse of the blanks in multivariate NMDS space were considered “indistinguishable.” Finally, we assessed the contribution of run-to-run variation by analyzing the effect of run in explaining variation in the microbial community of samples included on both runs. The effect of run was very small when compared to any other significant factor (100-fold smaller than any other significant factor at 0.02% variation explained, as determined by PERMANOVA tests, see below). From this we concluded that while there were differences attributable to run, the effect was negligible in the context of our questions. We retained only the duplicates that had been run on the second sequencing run for downstream analysis as that run had slightly better raw sequence quality.

### Statistical analyses

All statistical analyses were conducted in R [45] and data visualized using the *ggplot2* and *igraph* packages [46, 47]. We calculated pairwise community distances using the Bray-Curtis distance metric. We used PERMANOVA tests (R package *vegan*, [48]) to assess the degree to which variation in microbial community composition could be attributed to site and host species identity. We next tested whether the moss-associated microbial communities were structured by host phylogeny. In other words, we wanted to know if the differences between communities found in different host species are random with regards to the phylogenetic relatedness of the hosts. We used Mantel tests (R package *vegan*, [48]) to assess the degree of phylogenetic correlation between the host phylogeny and the composition of the microbial communities. Mantel tests have been shown to be effective measures of phylogenetic correlation in both real and simulated data and robust to community parameters such as the number of individuals in a community, the beta-diversity metric used, and the presence of microbe-microbe competition [49]. Mantel tests are also less susceptible to false-positive results when compared to phylogenetic congruence methods such as the Robinson-Foulds metric [49]. To contextualize our results, we also compared them to other studies that assessed phylogenetic signals in plant-associated microbial communities. We used the results of simulations from Mazel et al. [49] to determine the qualitative bin (strong, moderate, or weak) of each study’s results. Mazel et al. simulated microbial communities with different Blomberg’s *K* values ranging from weak to strong phylogenetic structuring and compared the values of Pearson’s *r* (Mantel test) corresponding to strong, moderate, and weak bins of Blomberg’s *K* values. Based on these results, we defined three bins of Pearson’s *r* values: *r* ≥ 0.5, strong; *r* ≥ 0.2 and *r* < 0.5, moderate; and *r* < 0.2, weak. Most studies used Mantel tests, but for those that did not, we marked the strength of the association as “unclear”.

To isolate the effects of site and moss species in our assessment of factors structuring microbial community differences, we subset the data in two ways: by site and by species. For the subsets by site, we divided the data by site and performed the analyses on the eight most moss-rich sites (i.e., the eight sites with the largest number of moss species, marked with an asterisk in Fig. 1). For the subsets by moss species, we focused on samples from those moss species which were most abundant and ubiquitous (Fig. 1). A moss species was defined as “abundant and ubiquitous” if it was found at all three Alaskan locations (i.e., Toolik, Fairbanks, and Anchorage) and had more than 10 samples collected in total (including replicates). The seven moss species meeting these criteria were *Sphagnum russowii*, *Aulacomnium palustre*, *Hylocomium splendens*, *Pleurozium schreberi*, *Ptilium crista*-*castrensis*, *Dicranum polysetum*, and *Polytrichum strictum*. This subset of moss species was also used for the analyses of putative N_2_-fixers (see below).

For each of the seven focal moss species, we used BIO-ENV analysis (R package *vegan*, [48]) to select the subset of environmental variables strongly correlated with bacterial community composition. Following this variable selection, we used multiple regression of distance matrices (MRM, R package *ecodist*, [50]) to determine which variables were significantly correlated (Spearman rank correlations) with the observed differences in microbial community composition.

### Identification of putative N_2_-fixers

To identify phylotypes that were well-correlated with measured N_2_-fixation rates, we ran Spearman correlations between N_2_-fixation rate (log-transformed) and the relative abundance of individual phylotypes. For each of the seven most abundant host species, we first filtered phylotypes for abundance and prevalence. Only those phylotypes with a mean relative abundance equal to or greater than 0.1% across all samples and those phylotypes that were present in at least three samples for a given host species were included in these analyses. We then identified phylotypes that were significantly positively correlated with N_2_-fixation rate as measured by FDR-corrected, Spearman correlations.

After identifying phylotypes that were positively correlated with N_2_-fixation, we attempted to independently verify whether these selected phylotypes were indeed likely capable of N_2_-fixation. A phylotype was identified as likely to possess the ability to fix N_2_ if it was (1) already known from the literature to fix N_2_ (e.g., members of *Nostocaceae*), (2) possessed a 16S rRNA gene more than 97% similar to an isolate able to grow on N-free media, or (3) if a publicly accessible metagenome-assembled genome (MAG) from a similar environment with at least a 97% similar 16S rRNA gene sequence also contained the *nifH* gene.

To identify closely related isolates capable of growth on N-free media, we used the RDP SeqMatch tool [51]. We identified MAGs from similar environments using publicly accessible MAGs from the Integrated Microbial Genomes and Microbiomes database (IMG, https://img.jgi.doe.gov/, [52]). We selected medium- and high-quality MAGs possessing a 16S rRNA gene that were categorized as coming from environments that were similar to those studied here. The environment categories (ontologies, as per MIMAG, [53]) were as follows: (1) host-associated, plants, peat moss (14 MAGs); (2) environmental, terrestrial, peat (174 MAGs); (3) environmental, terrestrial, soil, wetlands, permafrost (59 MAGs); and (4) environmental, terrestrial, unclassified, permafrost (293 MAGs). MAGs were accessed on August 30, 2019. For each putative N_2_-fixer, we identified any MAGs with a 16S rRNA gene at least 97% similar across our 253-bp PCR amplified gene region using the USEARCH global command [41]. For a full table of independently verified N_2_-fixers and the method of verification, see Table S5.

## Results

### Host structuring of bacterial community composition

**Fig. 2:**
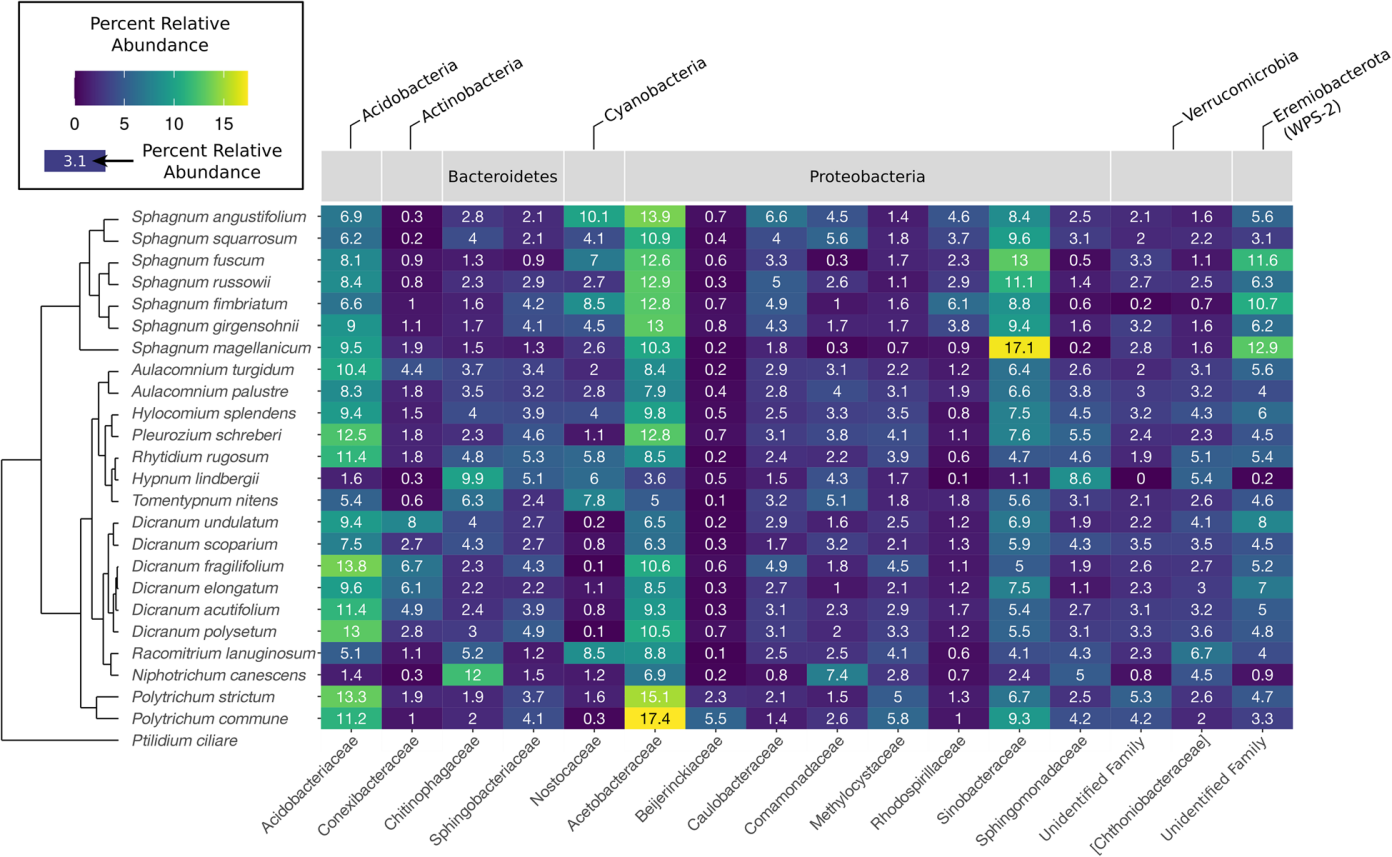
A heatmap showing the abundant bacterial families (*x*-axis) across different moss host species (*y*-axis) with a tree showing the phylogenetic relationships between the moss host species. Only 24 of the 26 moss species are included in this heatmap (see the “Methods” section). The outgroup host species, *Ptilidium ciliare*, is a liverwort. The colors indicate the relative abundance of each bacterial family with shades of yellow indicating higher relative abundance and shades of blue indicating lower relative abundance. Numbers in each cell of the heatmap show the percent relative abundance of a particular bacterial family

The moss-associated bacterial communities had an average richness of 812 unique phylotypes per sample and were dominated by eight bacterial phyla. The average relative abundances of these phyla across all samples were the following: *Proteobacteria* (49.6%), *Acidobacteria* (12.3%), *Actinobacteria* (8.4%), *Bacteroidetes* (7.5%), *Verrucomicrobia* (7.3%), *Candidatus Eremiobacterota* (WPS-2) (5.1%), *Planctomycetes* (4.2%), and *Cyanobacteria* (3.5%) (see Fig. 2 and Table S6 for details of family abundances across host species). Although the most abundant families and phylotypes found to be associated with the 26 moss species were fairly consistent across the samples, the relative abundances of these taxa varied depending on the host species in question. Differences in bacterial community composition were well-explained by host species identity and site (17.2 and 19.2 % of variation, respectively), with a significant portion of the variation explained by the interaction between site and host species (21.7 %) (Table 1).

**Table 1:** PERMANOVA results showing the relative importance of host identity and site in structuring the microbial communities of the 26 moss species

	Df	*F*	*R*^2^	*P*
Site	23	5.862	0.192	0.001
Species	25	4.829	0.172	0.001
Site to species	117	1.306	0.217	0.001
Total	-	-	0.581	-

A strong effect of host identity does not necessarily imply strong phylogenetic structuring; however, the differences in bacterial communities across the 26 host species were consistent with phylogenetic structuring as they were well-correlated with the phylogenetic distances between moss hosts (Mantel test, rho = 0.53, *p* = 0.001, Table S4). Much of this observed phylogenetic signal in moss bacterial community composition was driven by the unique communities associated with the *Sphagnum* mosses which themselves had a strong and significant phylogenetic signal when the analysis was restricted only to the seven S*phagnum* moss species (Mantel test, rho = 0.65, *p* = 0.005, Table S4).

Because moss species distributions are intrinsically linked to environmental conditions and not every species was present at every site, we used a subset of the samples to determine if the effects of host identity were still significant after controlling for site effects. To do this, we selected sites to test individually for the explanatory effect of host species, focusing only on those sites where greater than four moss species co-occurred with at least three individual samples collected per species from that site (for a total of eight sites that met the criteria) (Table S7). With site held constant, the variation explained by host species identity was significant at all eight sites and ranged from 29 (site 16, a white spruce upland) to 55% (site 5, a bog-like site dominated by black spruce) (Table S7). Together, these results indicate that there is a strong signal of both host identity and host phylogeny on moss bacterial community composition and this signal is independent of differences in site-level environmental conditions.

### Site-based drivers of bacterial community composition

In addition to the strong host taxonomic and phylogenetic signal in the composition of the moss bacterial communities, we also observed variation in bacterial community composition within a given host species across sites. In analyses of the seven most abundant and ubiquitous moss species, we found significant variation in bacterial communities attributable to site when host species was held constant (PERMANOVA, *R*^2^ = 0.37 to 0.62, *P* < 0.005 for all seven moss species, Table S8). To better understand the environmental factors that might help explain this variation attributable to the site differences, we determined which environmental variables were correlated with bacterial community composition (via a BIO-ENV analysis on each of the seven focal species) and then assessed the significance and strength of these variables (via MRM analyses) (Fig. S1). We identified between three and six environmental variables as important for structuring bacterial communities in each host species with total correlation values ranging from 0.36 (*Ptilium crista*-*castrensis*) to 0.60 (*Polytrichum strictum* and *Hylocomium splendens*). The most common predictors were site mean annual temperature, canopy cover, and percent hardwood (for the sites in this study, the hardwoods were all deciduous trees). These results suggest that temperature, light availability (as determined by canopy cover and tree type), and other canopy-based effects (such as litter depth) are important in structuring bacterial communities within individual moss species. However, when we tested these variables for significance with MRM, most predictors had small coefficient values (< 0.9) and the subset of significant predictors varied greatly between host species. Thus, although some of the variation in the composition of the bacterial communities is associated with light availability and temperature, the importance of these effects was generally small and highly variable depending on the moss species in question.

### Identifying potential N_2_-fixers found in boreal mosses

**Fig. 3:**
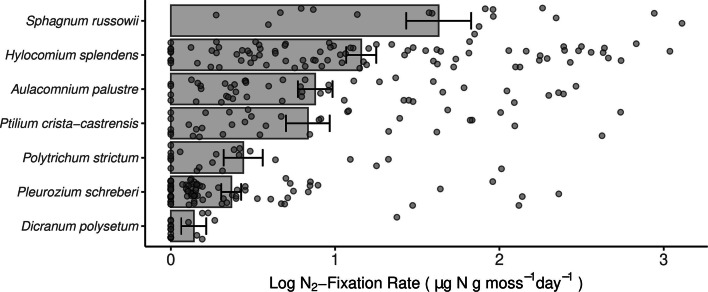
N_2_-fixation rates for the seven abundant and ubiquitous species in our data set as measured by isotope enrichment over a 1-day incubation period. Moss species are arranged from highest average fixation rate to lowest. All seven species hosted N_2_-fixing microbes however rates were highly variable across host species. Each circle represents the measured fixation rate in one moss sample. Error bars represent 1 standard error above and below the mean (which is indicated by the height of the main bar). A more in-depth analysis of the differences in N_2_-fixation across moss species can be found in Stuart et al. [31]

**Fig. 4:**
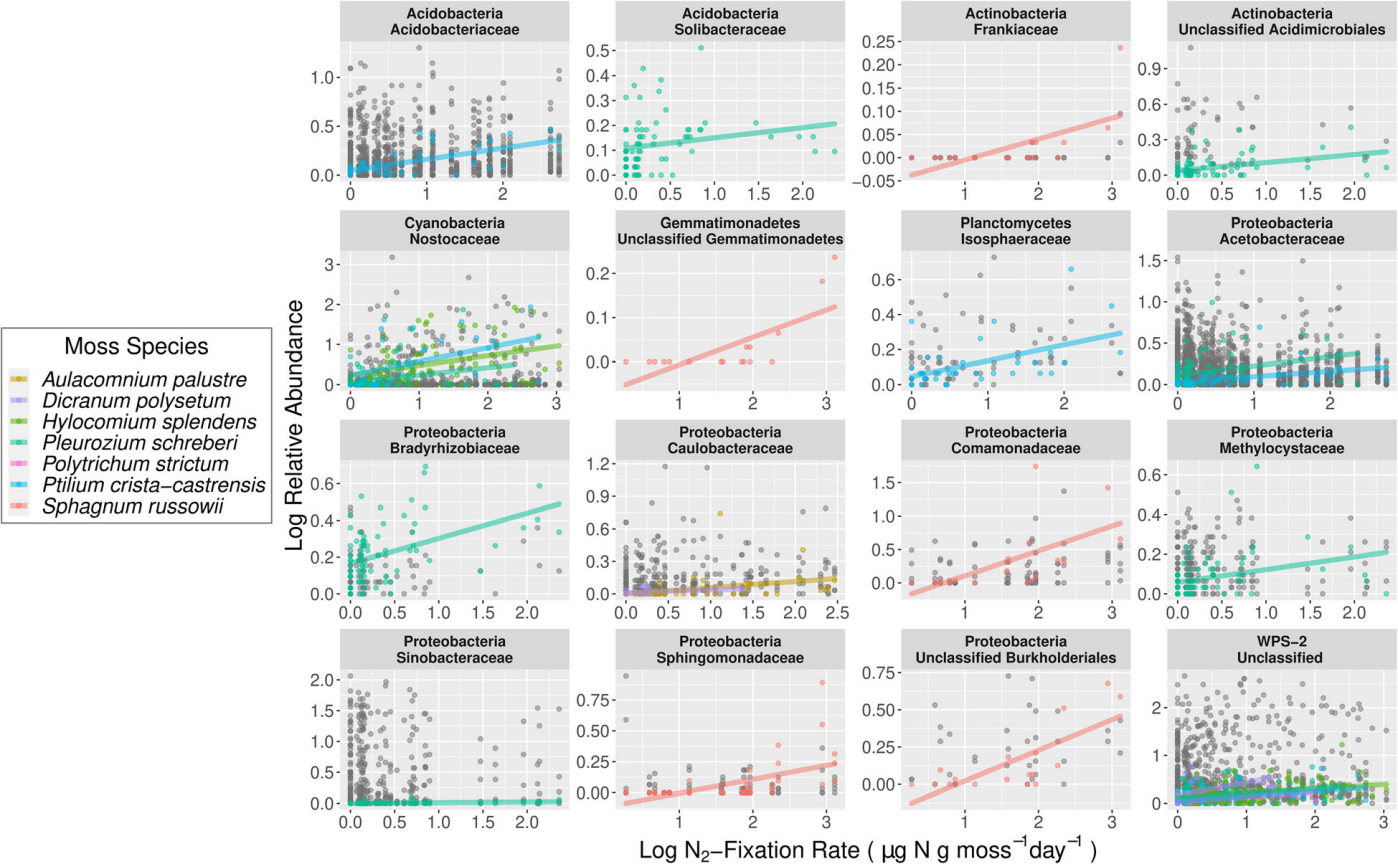
Spearman correlations between the relative abundances of individual bacterial phylotypes and measured N_2_-fixation rates. Bacterial phylotypes used in this analysis were limited to those that had an average relative abundance of > 0.1% across the more abundant and ubiquitous moss host species (156 phylotypes). The data is separated into panels based on the family-level taxonomy of the phylotypes. Colored points and lines represent bacterial phylotypes that were significantly positively correlated with N_2_-fixation in a particular moss species. Although not indicated on the plot for clarity, correlation coefficients for each phylotype can be found in Supplementary Table S6. Lines and points are colored according to the moss species in question. Gray dots are measurements for 137 phylotypes that were not found to be significantly positively correlated with N_2_-fixation rates. We note that, to improve clarity, the *x* and *y* scales vary between panels

**Fig. 5:**
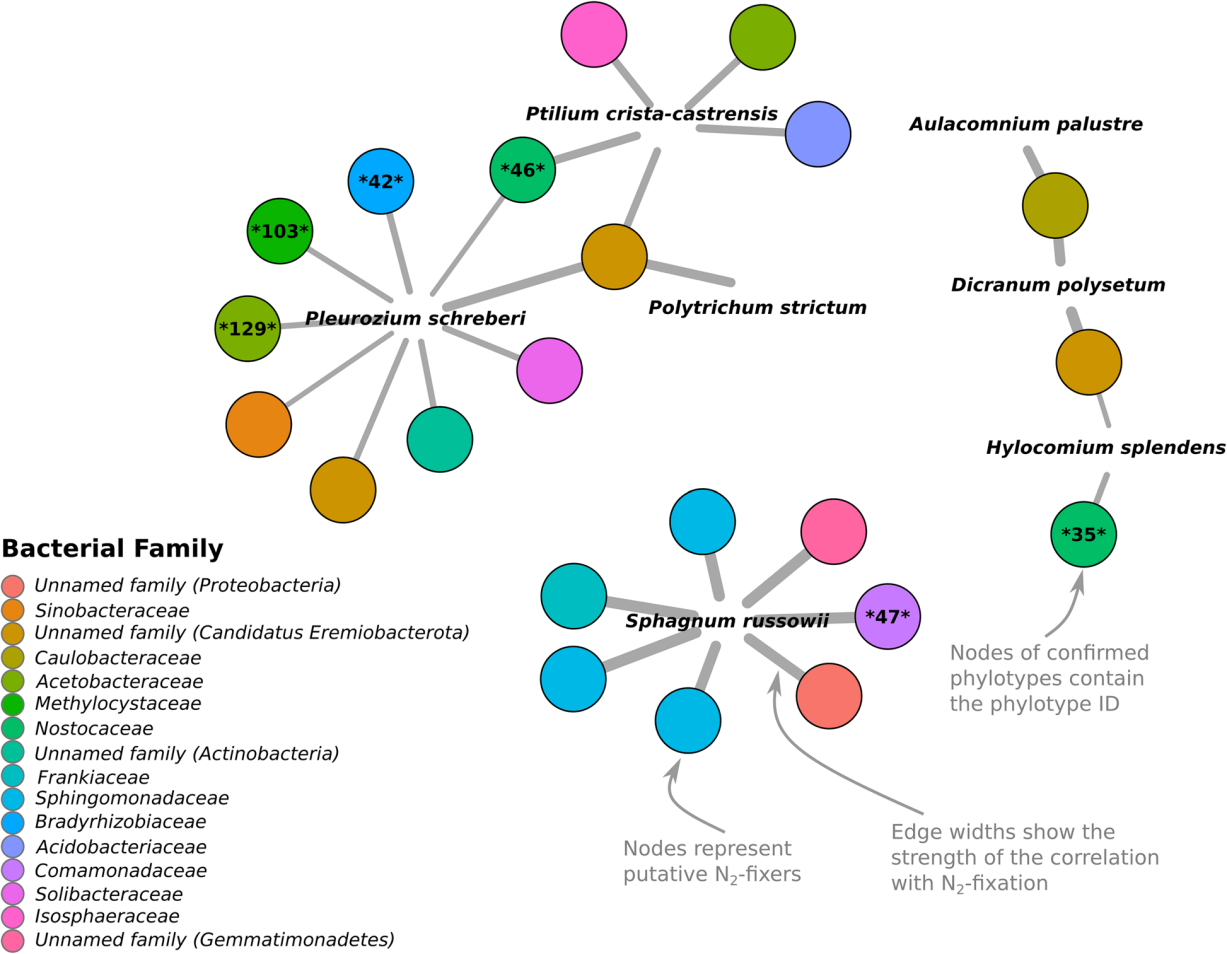
Correlation network showing phylotypes (represented by colored nodes) that had significant positive Spearman correlations with N_2_-fixation rates (rho, 0.33–0.78; *P* < 0.05, see Supplementary Table S6 for details) within each of the abundant moss species. Colors of the nodes represent the family membership of each phylotype. Phylotypes that were confirmed as potential N_2_-fixers are labeled with their unique identifier and asterisks (see Supplementary Table S5 for details)

Measured rates of N_2_-fixation ranged from 0 to 21.45 μg N g moss dry wt.^−1^ day^−1^ (across the 301 samples collected from the seven focal moss species that were abundant and ubiquitous in our sample set (Fig. 3)). To identify those bacterial taxa that were associated with higher N_2_-fixation rates across the seven focal species, we ran Spearman correlations between the relative abundances of taxa and log-transformed N_2_-fixation rates. We identified 16 bacterial families containing 22 phylotypes that were positively correlated with measured N_2_-fixation rates (Fig. 4). Correlations ranged from 0.34 (phylotype 33—*Sinobacteraceae*) to 0.75 (phylotype 94—*Sphingomonas echinoides*). The bacterial families with relative abundances positively correlated with measured N_2_-fixation rates included both taxa known to fix N_2_ in mosses (i.e., taxa from the cyanobacterial family *Nostocaceae*) and those which have not yet been identified as capable of N_2_-fixation (i.e., taxa from *Acidobacteraceae*). Interestingly, the phylotypes found to be positively associated with measured N_2_-fixation rates were not necessarily consistent between moss hosts. To better visualize these associations, we created an association network of the correlations (Fig. 5). While most phylotypes were significantly correlated with N_2_-fixation rates across multiple moss hosts, *S*. *russowii* hosted a unique set of taxa. Furthermore, although most mosses were associated with taxa from a range of bacterial lineages, all of the phylotypes significantly associated with *S*. *russowii* were from the phylum *Proteobacteria*.

The correlations between the relative abundances of individual bacterial taxa and measured N_2_-fixation rates (shown in Fig. 3) do not necessarily indicate that those bacterial taxa are actually capable of N_2_-fixation. Thus, we used several approaches to try to confirm the N_2_-fixation capabilities of the bacterial taxa that were correlated with measured N_2_-fixation rates. A phylotype was identified as likely to possess the ability to fix N_2_ if it was already known from the literature to fix N_2_ (e.g., members of *Nostocaceae*), if it was closely related to a previously isolated bacterial strain able to grow on N-free media, or if a highly similar metagenome-assembled genome (MAG) obtained from a similar environment also contained the *nifH* gene. Using these methods, six of the 22 phylotypes were confirmed as likely to possess the ability to fix N_2_ (Fig. 5 and Table S5). Notably, only two of the six confirmed phylotypes belong to the family *Nostocaceae*, an N_2_-fixing clade known to associate with mosses. The other four phylotypes come from the family *Methylocystaceae* and the genera *Bradyrhizobium*, *Methylibium*, and *Acidisoma*, expanding the list of bacterial taxa which are likely capable of N_2_-fixation in boreal mosses. For the other 16 phylotypes, there was not enough evidence to determine their N_2_-fixation ability (although some of these phylotypes came from clades where N_2_-fixation is common such as *Burkholderiales*, *Frankiaceae*, or *Acetobacteraceae*). Finally, several phylotypes, such as the three from the phylum *Candidatus Eremiobacterota* (formerly WPS-2) are unlikely to be N_2_-fixers as no genomes from members of this phylum have been shown to contain the *nifH* gene. In total, we can only confirm that 6 of the 22 bacterial phylotypes identified through our correlation-based analyses (Fig. 5) are likely capable of N_2_-fixation.

## Discussion

### The role of host phylogeny in shaping moss bacterial communities

A primary goal of this study was to determine the relative importance of host identity versus site in structuring moss microbial communities. Here, we show that moss-associated bacterial communities were strongly structured by host identity and host phylogeny across the 26 moss species surveyed. These results are consistent with previous studies that focused on smaller subsets of moss species which have also shown that distinct moss host species harbor distinct bacterial communities [15, 16, 54]. Our study extends these results by encompassing a broader diversity of moss species sampled across a large geographic area. We also show that a significant portion of the observed variation in bacterial community composition can be attributed to the phylogenetic relationships between moss species and that this phylogenetic structure is largely attributable to the *Sphagnum* mosses harboring distinct bacterial communities. This phylogenetic signal in the microbiome may be the result of co-diversification between a host and its microbial inhabitants, but it is more likely a product of an independent phylogenetically structured trait (or traits) selecting for certain microbes from the environment (“ecological filtering”, [49, 55]). Importantly, the phylogenetic signal we observed is stronger than simulations suggest should be expected for an ecological filtering model [49]. This suggests that either there has been co-diversification of the bacterial communities with their moss hosts (unlikely given the divergence of *Sphagnum* from other mosses is close to 400 mya, [56]) or that the moss traits that are responsible for the ecological filtering of bacterial communities exhibit a phylogenetic signal that is stronger than is typically expected (i.e., Blomberg *K* >>1, [57]).

This strong phylogenetic signal in the structure of the moss microbiome stands in stark contrast to most vascular plants which typically exhibit no signal or very weak phylogenetic signals between host species (for a summary of available literature, see Table S9). However, the majority of studies investigating phylogenetic signals in plant microbiomes focus on rhizosphere communities. The patterns for aboveground bacterial communities in plants are less well understood. Although previous studies have found that plant species have distinct phyllosphere communities (reviewed in [58–60]), we know of only three studies that have explicitly tested for a phylogenetic signal in these communities. Across these studies, all of which focused on vascular plants (and trees, in particular), the strength of phylogenetic signal ranges from moderate to strong [61–63]. Although direct comparisons are challenging, the strength of the phylogenetic signal in moss microbial communities is as strong or stronger than any of these previous phyllosphere studies and particularly strong when taken in the context of the plant microbiome literature as a whole.

The observed phylogenetic signal in microbiome composition is strongly driven by *Sphagnum* mosses harboring bacterial communities distinct from those found in other mosses. The strong phylogenetic signal in the *Sphagnum* mosses’ bacterial communities could be due to ecological filtering by the internal compartments (i.e., hyaline cells) that are unique to *Sphagnum* anatomy. Hyaline cells hold moisture and harbor microbes [13] and attributes of these cells vary among *Sphagnum* species [64]. Internal compartments are often linked with stronger phylogenetic signals in other host microbiomes [49, 55]. *Sphagnum* species also acidify their local environment [64] and induce changes in canopy cover and moisture availability [2]—factors that could effectively select for the unique microbial communities associated with *Sphagnum* mosses.

### Diverse potential N_2_-fixing taxa in moss bacterial communities

Mosses in northern latitude ecosystems harbor a diverse pool of potential N_2_-fixers, which play a key role in regulating N availability. We were able to identify 22 bacterial phylotypes from 14 bacterial families that had relative abundances positively correlated with measured N_2_-fixation rates (Fig. 4), pointing to strong associations between a diverse group of bacterial taxa and measured N_2_-fixation rates. Moss species host distinct communities of potential N_2_-fixers with a different set of phylotypes correlating more strongly with each host species (Fig. 5). Members of the *Sphagnum* genus (particularly *S*. *russowii*) generally hosted a unique set of bacterial taxa that was not associated with N_2_-fixation rates in any of the other moss species sampled (Fig. 5). *Sphagnum* species not only host unique, phylogenetically structured microbial communities (Fig. 2, Table S4), they also host a unique community of potential N_2_-fixers and this unique community may partly explain the high N_2_-fixation rates observed across the *Sphagnum* species (Fig. 3).

Our results suggest that N_2_-fixation may be carried out by a number of bacterial taxa beyond those within the phylum *Cyanobacteria* (Fig. 5), which are often considered the predominant N_2_-fixers in mosses [4, 23]. While abundance and N_2_-fixation are not necessarily linked and even low abundance N_2_-fixers can contribute to high N_2_-fixation rates [7], our hypothesis that there are more non-cyanobacterial N_2_-fixers in boreal mosses than previously recognized is in line with previous studies in *Sphagnum* mosses [26, 27]. Our results support these findings and broaden them to include other moss lineages since the phylotypes most strongly associated with fixation rates on *Sphagnum russowii* were non-cyanobacterial and each of our seven focal moss species also hosted multiple putative N_2_-fixers that were non-cyanobacterial.

The potential for mosses to harbor a broad diversity of N_2_-fixing bacteria is likely an important ecological feature. While it is possible that distinct N_2_-fixers may be in direct competition [54], it is also possible that they could have distinct niches within the moss host. For example, oxygenic photosynthesizers, such as cyanobacteria, may be limited by light conditions, while anoxygenic photosynthesizing N_2_-fixers, such as (members of the genus *Acidisoma*, [65]) may be able to fix N_2_ in light conditions that are sub-optimal for cyanobacteria [66]. A promising direction for future work is to determine how N_2_-fixation rates in mosses are the product of interactions (direct or indirect) between the diverse array of N_2_-fixers that can be found in a given host (e.g., [67]).

### The role of environment in shaping moss bacterial communities

In addition to distinct species and lineages of mosses having unique bacterial communities, the bacteria associated with individual moss species were variable in composition and some of this intra-species variation in bacterial community composition could be predicted from the measured site characteristics, namely light availability and temperature. Light availability is particularly interesting as it is a commonly cited factor governing N_2_-fixation rates [11, 68, 69]. Multiple studies have attributed light-induced increases in N_2_-fixation to the presence of photosynthetic N_2_-fixing cyanobacteria. However, other phototrophic organisms (including anoxygenic phototrophs from *Alphaproteobacteria* or the candidate phylum *Eremiobacterota* (WPS-2)) are also prominent members of moss bacterial communities [15], and these taxa may also be contributing to the bacterial community responses to light availability, highlighting the potentially important contributions of phototrophs in the moss microbiome. This finding is important as temperature and light availability are some of the more variable environmental conditions in high-latitude systems and both factors are likely to shift with climate change due to elevated temperatures and as denser-canopied deciduous birch (*Betula*) species encroach on formerly open-canopied spruce-dominated areas with increases in fire prevalence [70–72].

Since a large portion of variation is attributable to site, but the environmental predictors we measured were fairly weak predictors, it is likely that “site” may be better represented by a suite of factors not measured in this study. These factors might include abiotic factors such as stand age, parent bedrock material, and even trace metal availability [4, 73] or local biotic factors such as host moss genotype, changes in light availability through shading by understory vascular plant communities (not captured in our measurements of canopy cover), top-down controls by protists and microfauna [74, 75], or community turnover due to microbial colonization from the surrounding local micro-environment [76]. Regardless, our results suggest that host species identity, rather than measured or unmeasured environmental factors, is a more consistent indicator of bacterial community structure and that moss species distributions may be more relevant than site characteristics for understanding the contributions of the moss microbiome to ecosystem processes, including N_2_-fixation.

## Conclusions

The results from this study indicate that microbiomes of boreal mosses are phylogenetically structured and that moss species identity, not site environmental conditions, is the best predictor of microbial community composition. Conversely, moss microbial communities are not easily predictable from site characteristics, although our results suggest that light and temperature can have significant, though subtle, effects on the composition of the moss microbiome. We also found that the identities of the potential N_2_-fixing bacteria were host species-specific suggesting that N_2_-fixation may be best predicted at the host-level. Finally, many of the potential N_2_-fixing microbes we identified were not cyanobacteria. These non-cyanobacterial bacteria are worthy of future study to better understand the processes controlling fixation rates in boreal mosses. More generally, our work demonstrates the utility of mosses for studying plant microbiomes and broadens our understanding of how moss-microbe interactions contribute to N dynamics in high-latitude ecosystems.

## Supplementary Information

**Additional file 1: Table S1.** A list of all of the moss species sampled and the number of samples collected for each. **Table S3.** List of sites and site characteristics. 10 sites around Fairbanks, AK, USA, and four sites around Toolik Lake, AK, USA were sampled in 2016 and an additional 10 sites from around Anchorage, AK, USA, were added in 2017. Sites were chosen to capture a breadth of common habitat types in boreal and tundra ecosystems. **Table S6.** Table summarizing the results of a Mantel test between moss microbial communities and moss phylogenetic relatedness. Microbial communities are significantly correlated with phylogenetic structure. An analysis of *Sphagnum* shows that these results are primarily driven by phylogenetic structuring within the *Sphagnum* branch of the phylogeny. **Table S7.** Results of PERMANOVAs run on eight individual sites (a subset of the 24 total sites that had >4 moss species present in >3 replicates) showing the effect of species in explaining microbial community differences. **Figure S1.** The best environmental predictors shaping microbial communities for each of the seven most abundant host species. **Table S8.** Results of PERMANOVAs run on seven individual species showing the effect of site in explaining microbial community differences.

**Additional file 2: Table S2.** A list of all of the moss species sampled and where and how many samples were collected for each. The species sampled include 26 moss species.

**Additional file 3: Table S4.** Summary of the evidence for N_2_-fixation for each of the 22 phylotypes positively correlated with N_2_-fixation rate.

**Additional file 4: Table S5:** Relative abundances of microbial families in each host species.

**Additional file 5: Table S9:** Table summarizing the correlations between N_2_-fixation rate and the 22 putative N_2_-fixing phylotypes. We note that some phylotypes are listed more than once as they had significant positive correlations in multiple host species.

**Additional file 6: Table S10:** Table summarizing the findings from nine studies about prevalence and strength of host phylogenetic structuring of plant-microbial communities.

## Data Availability

The datasets generated and analyzed during the current study are available in the GenBank Sequence Read Archive (SRA), accession number: PRJNA622832 https://www.ncbi.nlm.nih.gov/bioproject/PRJNA622832. Under this identifier, the raw, unprocessed reads and all associated environmental data used in this study are available for each sample. The permanent identifiers for each genome used from IMG as evidence for nitrogen-fixing potential can be found in Table S4. IMG genomes can be accessed at: https://img.jgi.doe.gov/
