# Supplementary material for: The bacterial communities of Alaskan mosses and their contributions to N2-fixation
Source: Microbiome. 2021 Feb 23;9:53. doi: 10.1186/s40168-021-01001-4 (PMC7903681; doi:10.1186/s40168-021-01001-4)
Supplement: Supplementary file 2 — Additional file 1: Table S1. A list of all of the moss species sampled and the number of samples collected for each. Table S3. List of sites and site characteristics. 10 sites around Fairbanks, AK, USA, and four sites around Toolik Lake, AK, USA were sampled in 2016 and an additional 10 sites from around Anchorage, AK, USA, were added in 2017. Sites were chosen to capture a breadth of common habitat types in boreal and tundra ecosystems. Table S6. Table summarizing the results of a Mantel test between moss microbial communities and moss phylogenetic relatedness. Microbial communities are significantly correlated with phylogenetic structure. An analysis of Sphagnum shows that these results are primarily driven by phylogenetic structuring within the Sphagnum branch of the phylogeny. Table S7. Results of PERMANOVAs run on eight individual sites (a subset of the 24 total sites that had >4 moss species present in >3 replicates) showing the effect of species in explaining microbial community differences. Figure S1. The best environmental predictors shaping microbial communities for each of the seven most abundant host species. Table S8. Results of PERMANOVAs run on seven individual species showing the effect of site in explaining microbial community differences. [file 40168_2021_1001_MOESM2_ESM.docx]

## Supporting Information

Article title: The bacterial communities of Alaskan mosses and their contributions to N_2_-fixation

Authors: Hannah Holland-Moritz, Julia E. M. Stuart, Lily R. Lewis, Samantha N. Miller, Michelle C. Mack, Jose Miguel Ponciano, Stuart F. McDaniel, Noah Fierer

Article acceptance date: NA

List of supplementary tables and figures and their locations

**Table S1** A list of all of the moss species sampled and the number of samples collected for each.

**Table S2** A list of all of the moss species sampled and where and how many samples were collected for each. The species sampled include 26 moss species. (Submitted separately)

**Table S3** List of sites and site characteristics. 10 sites around Fairbanks, AK, USA, and four sites around Toolik Lake, AK, USA were sampled in 2016 and an additional 10 sites from around Anchorage, AK, USA, were added in 2017. Sites were chosen to capture a breadth of common habitat types in boreal and tundra ecosystems

**Table S4** Summary of the evidence for N_2_-fixation for each of the 22 phylotypes positively correlated with N_2_-fixation rate. (Submitted separately)

**Table S5** Relative abundances of microbial families in each host species. (Submitted separately)

**Table S6** Table summarizing the results of a Mantel test between moss microbial communities and moss phylogenetic relatedness. Microbial communities are significantly correlated with phylogenetic structure. An analysis of Sphagnum shows that these results are primarily driven by phylogenetic structuring within the Sphagnum branch of the phylogeny.

**Table S7** Results of PERMANOVAs run on eight individual sites showing the effect of species in explaining microbial community differences.

**Fig. S1** The best environmental predictors shaping microbial communities for each of the seven most abundant host species.

**Table S8** Results of PERMANOVAs run on seven individual species showing the effect of site in explaining microbial community differences.

**Table S9** Table summarizing the correlations between N_2_-fixation rate and the 22 putative N_2_-fixing phylotypes. (Submitted separately)

**Table S10** Table summarizing the findings from nine studies about prevalence and strength of host phylogenetic structuring of plant-microbial communities. (Submitted separately)

**Table S1** A list of all of the moss species sampled and the number of samples collected for each. The species sampled include 26 moss species and one liverwort (used as a phylogenetic outgroup).

| **Moss Species** | **Number of Samples** |
| --- | --- |
| *Hyocomium splendens* (Hedw.) Schimp. | 87 |
| *Pleurozium schreberi* (Brid.) Mitt | 80 |
| *Aulacomnium palustre* (Hedw.) Schwägr. | 47 |
| *Ptilium crista-castrensis* (Hedw.) De Not. | 36 |
| *Polytrichum commune* Hedw. | 27 |
| *Polytrichum strictum* Brid. | 22 |
| *Sphagnum russowii* Warnst. | 19 |
| *Dicranum polysetum* Sw. | 19 |
| *Aulacomnium turgidum* (Wahlenb.) Schwägr. | 16 |
| *Dicranum acutifolium* (Lindb. & Arnell) C.E.O. Jensen ex Weinm. | 12 |
| *Sphagnum girgensohnii* Russow | 10 |
| *Sphagnum angustifolium* (C.E.O. Jensen ex Russow) C.E.O. Jensen | 10 |
| *Rhytidium rugosum* (Hedw.) Kindb. | 9 |
| *Sphagnum magellanicum* Brid. | 9 |
| *Tomentypnum nitens* (Hedw.) Loeske | 8 |
| *Dicranum scoparium* Hedw. | 8 |
| *Dicranum undulatum* Brid. | 7 |
| *Dicranum elongatum* Schleich. ex Schwägr. | 7 |
| *Sphagnum fuscum* (Schimp.) Klinggr. | 6 |
| *Dicranum fuscescens* Turner | 5 |
| *Dicranum fragilifolium* Lindb. | 5 |
| *Racomitrium lanuginosum* (Hedw.) Brid. | 4 |
| *Sphagnum squarrosum* Crome | 4 |
| *Niphotrichum canescens* (Hedw.) Bednarek-Ochyra & Ochyra | 3 |
| *Sphagnum fimbriatum* Wilson | 1 |
| *Hypnum lindbergii* Mitt. | 1 |
|  |  |
| **Liverwort (used as outgroup)** |  |
| *Ptilidium ciliare* (L.) Hampe | 9 |

**Table S2** A list of all of the moss species sampled and where and how many samples were collected for each. The species sampled include 26 moss species. (Table provided in a separate file)

**Table S3** List of sites and site characteristics. 10 sites around Fairbanks, AK, USA, and four sites around Toolik Lake, AK, USA were sampled in 2016 and an additional 10 sites from around Anchorage, AK, USA, were added in 2017. Sites were chosen to capture a breadth of common habitat types in boreal and tundra ecosystems.

| **Site ID** | **Latitude** | **Longitude** | **Focal Transect Location** | **Site Description** |
| --- | --- | --- | --- | --- |
| 1 | 64.77 | -148.3 | Fairbanks | Bonanza Creek, *Picea mariana* (Black Spruce) dominant |
| 2 | 64.77 | -148.27 | Fairbanks | Bonanza Creek, *Picea mariana* dominant |
| 3 | 64.77 | -148.3 | Fairbanks | Bonanza Creek, *Picea mariana* dominant |
| 4 | 64.71 | -148.31 | Fairbanks | Bonanza Creek *Picea glauca*/Alder/Birch dominant |
| 5 | 64.7 | -148.3 | Fairbanks | Bonanza Creek bog-like, *Picea mariana* dominant |
| 6 | 64.7 | -148.29 | Fairbanks | Bonanza Creek, fen-like, open canopy |
| 7 | 64.87 | -147.86 | Fairbanks | UAF, tussock |
| 8 | 64.96 | -148.37 | Fairbanks | Murphy Dome, Montane tundra |
| 9 | 64.88 | -148.39 | Fairbanks | Murphy Dome, Aspen dominated |
| 10 | 64.88 | -148.4 | Fairbanks | Murphy Dome, *Picea mariana* dominant |
| 11 | 68.64 | -149.59 | Toolik Lake | Toolik, heath, many rocks no canopy |
| 12 | 68.64 | -149.57 | Toolik Lake | Toolik, shrub tundra, no canopy |
| 13 | 68.64 | -149.57 | Toolik Lake | Toolik, moist acidic tundra, no canopy |
| 14 | 68.63 | -149.64 | Toolik Lake | Toolik, basic tundra, no canopy |
| 15 | 61.2 | -149.81 | Anchorage | UAA Arboretum, *Picea mariana* dominant, bog-ish |
| 16 | 61.2 | -149.81 | Anchorage | UAA Arboretum, *Picea glauca* (White Spruce) upland |
| 17 | 61.16 | -149.8 | Anchorage | Campbell Creek, hummocky mixed dominance forest |
| 18 | 61 | -149.09 | Anchorage | Winner's Creek, Girdwood, *Tsuga heterophylla* (western hemlock) forest |
| 19 | 60.97 | -149.11 | Anchorage | Moose Meadows, Girdwood, *Tsuga heterophylla*/ *Picea* dominant |
| 20 | 60.97 | -149.11 | Anchorage | Moose Meadows, Girdwood, open fen-like |
| 21 | 61.14 | -149.77 | Anchorage | Anchorage High School (AC), *Picea mariana* dominant |
| 22 | 61.17 | -149.78 | Anchorage | Campbell Creek (AB), *Picea mariana* dominant |
| 23 | 61.2 | -149.81 | Anchorage | UAA Arboretum (AA), *Picea mariana* dominant |
| 24 | 61.22 | -149.43 | Anchorage | Harp Mountain, Alpine tundra, *Betula nana* |

**Table S4** Evidence for N_2_-fixation for each of the 22 phylotypes positively correlated with N_2_-fixation rate. For each phylotype a we specify whether or not there is evidence for N_2_-fixation, and if there is evidence, we specify the type of evidence (literature, isolate genome capable of growth on N-free media, or metagenome). For each phylotype, we also list the closest isolated relative (measured using RDP’s SeqMatch tool) and provide the similarity score for that isolate. For phylotypes with matches among the publicly-accessible MAGs from similar environments, the IMG metagenome bin ID is listed and the 16S SSU distances (as measured by the USEARCH global command) to the phylotype with >0.90 similarity scores are provided. In addition to SSU similarity information, we provide the percent completeness, percent contamination, 16S SSU IMG gene ID, and *nifH* IMG gene ID for MAGs that provide evidence for N_2_-fixation. In cases where literature was used to support a claim of N_2_-fixation or growth on N-free media, the relevant citation is provided. (Table provided in a separate file)

**Table S5** Relative abundances of microbial families across host species. Only families that were present at greater an average relative abundance of 0.05 were included in the analysis. Host species are arranged in alphabetical order. Due to the large number of host species, the table has been split into three parts for readability. Significance values represent the results of Bonferroni-corrected p-values from a Kruskall-Wallace test of significance differences in the bacterial family’s relative abundance between host species. All families were found to significantly differ between hosts. (Table provided in a separate file)

**Table S6** Table summarizing the results of a Mantel test between moss microbial communities and moss phylogenetic relatedness. Microbial communities are significantly correlated with phylogenetic structure. An analysis of Sphagnum shows that these results are primarily driven by phylogenetic structuring within the Sphagnum branch of the phylogeny.


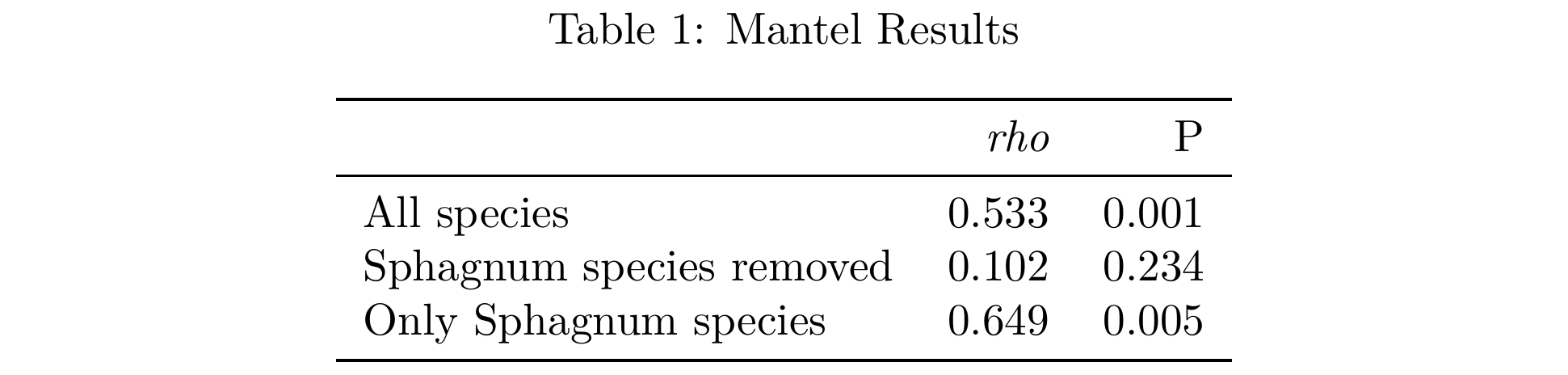


**Table S7** PERMANOVA run on eight individual sites showing the effect of species in explaining microbial community differences.


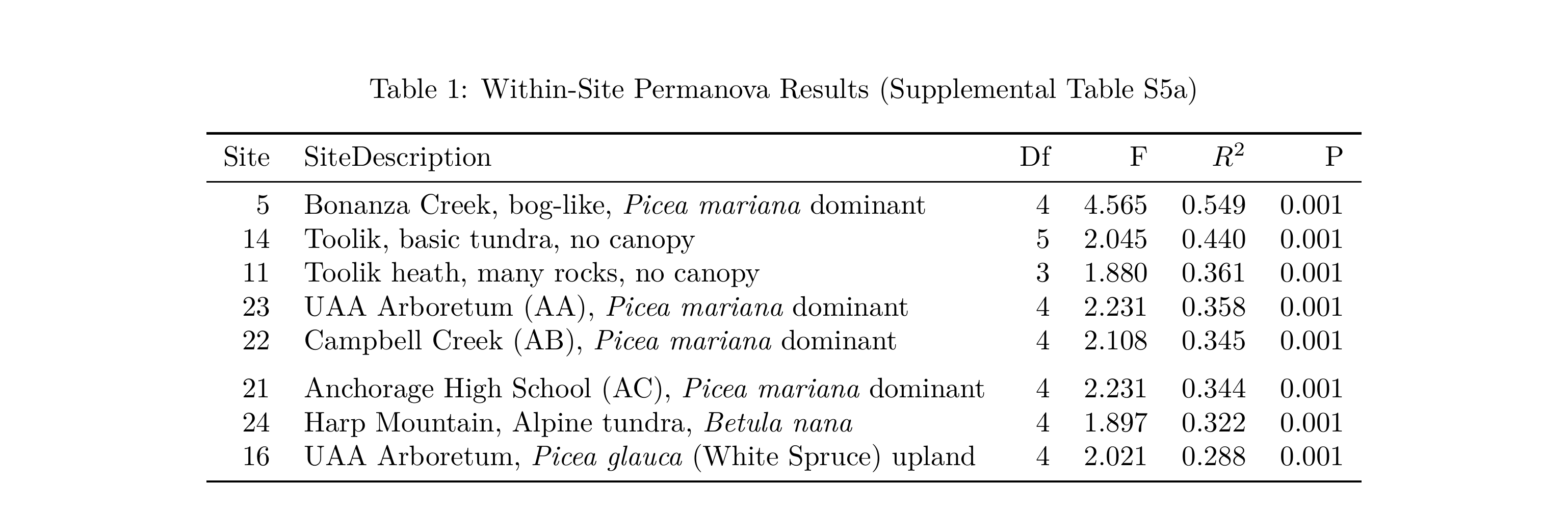


**Fig. S1** Best environmental predictors shaping microbial communities on seven most abundant host species. Green boxes indicate the best predictors as chosen by BIO-ENV for each host species. Rho values for the BIO-ENV tests are displayed beneath the species names. Text inside the boxes indicate the results of a multiple regression of distance matrices (MRM), the top number indicates the regression coefficient, the bottom represents the p-value for that predictor. Darker green boxes indicate a coefficient that was confirmed significant in the MRM. Lighter green boxes were not significant. All correlations for these tests were measured with spearman rank correlations.


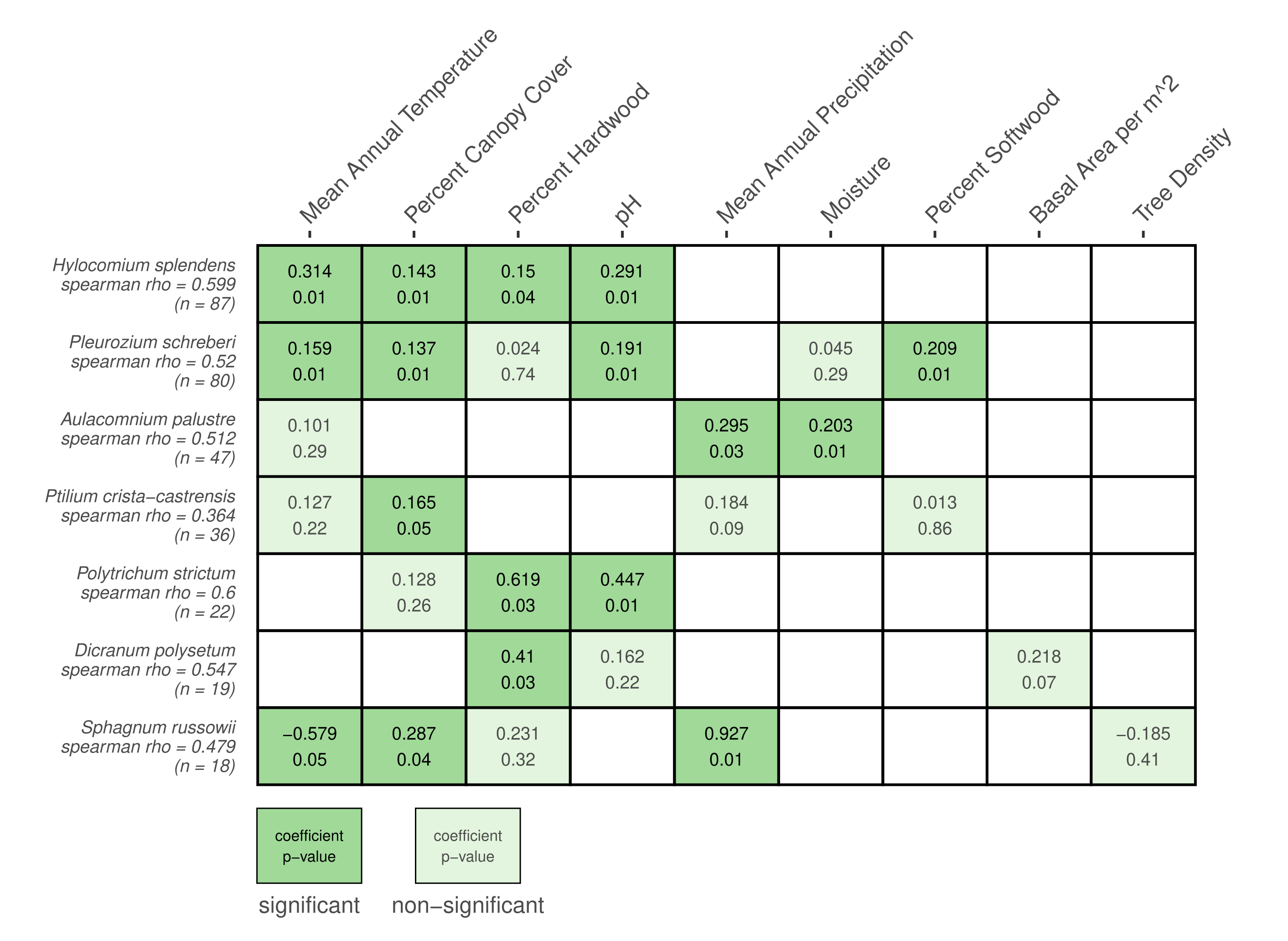


**Table S8** PERMANOVA run on seven individual species showing the effect of site in explaining microbial community differences.

**
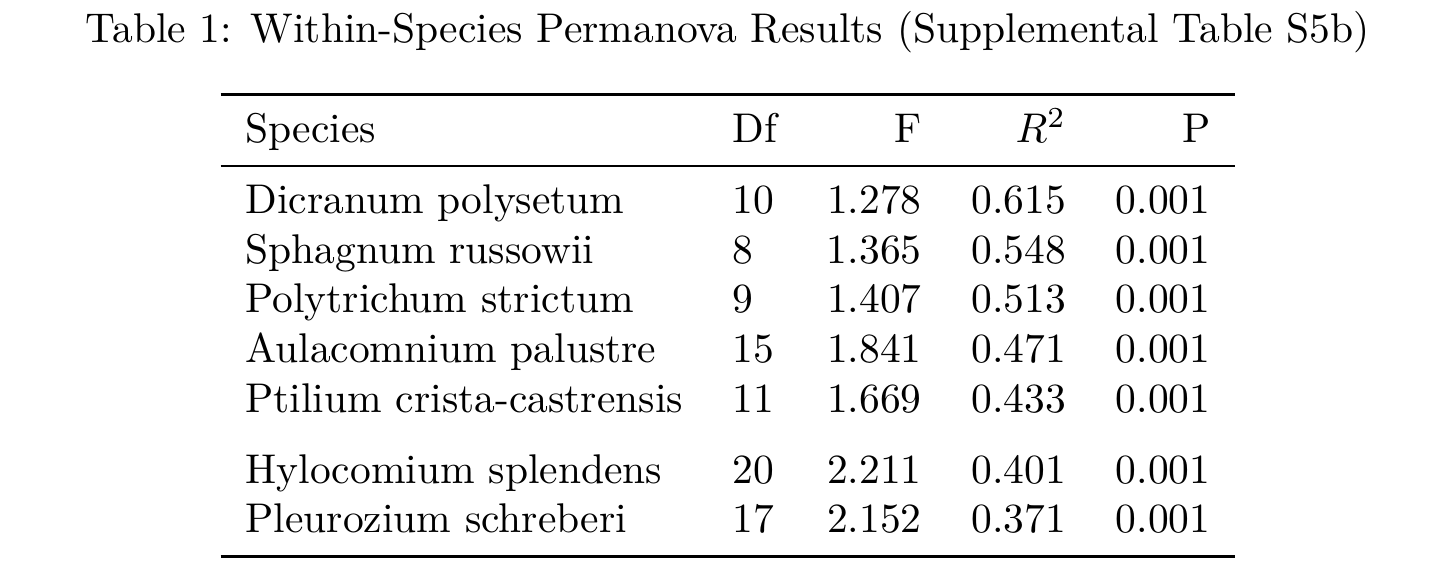
**

**Table S9** Table summarizing the correlations between N_2_-fixation rate and the 22 putative N_2_-fixing phylotypes. We note that some phylotypes are listed more than once as they had significant positive correlations in multiple host species. (Table provided in separate file)

**Table S10** Table summarizing the findings from nine studies about prevalence and strength of host phylogenetic structuring of plant-microbial communities. The strength quality column is based on theoretical expectations from simulated communities ref [44]. (Table provided in separate file)
